# Differential Effects of Low- and High-Dose Dexamethasone on Electrically Induced Damage of the Cultured Organ of Corti

**DOI:** 10.1007/s12640-020-00228-7

**Published:** 2020-06-03

**Authors:** Marvin N. Peter, Gerrit Paasche, Uta Reich, Thomas Lenarz, Athanasia Warnecke

**Affiliations:** 1 Department of Otorhinolaryngology, Head and Neck Surgery, Hannover Medical School, Carl-Neuberg-Str. 1, 30625 Hannover, Germany; 2 Cluster of Excellence “Hearing4all” of the German Research Foundation, Hannover, Germany; 3 Department of Otorhinolaryngology, Head and Neck Surgery, Berlin Institute of Health, Charité-Universitätsmedizin Berlin, Humboldt-Universität zu Berlin, Berlin, Germany

**Keywords:** Hearing loss, Electric stimulation, Dexamethasone, Organ of Corti, Reactive oxygen species, Synapses

## Abstract

An increased number of patients with residual hearing are undergoing cochlear implantation. A subset of these experience delayed hearing loss post-implantation, and the aetiology of this loss is not well understood. Our previous studies suggest that electrical stimulation can induce damage to hair cells in organ of Corti (OC) organotypic cultures. Dexamethasone has the potential to protect residual hearing due to its multiple effects on cells and tissue (e.g., anti-inflammatory, free radical scavenger). We therefore hypothesized that dexamethasone treatment could prevent electrical stimulation induced changes in the OC. Organ of Corti explants from neonatal rats (P2–4) were cultured for 24 h with two different concentrations of dexamethasone. Thereafter, OC were subjected to a charge-balanced biphasic pulsed electrical stimulation (0.44–2 mA) for a further 24 h. Unstimulated dexamethasone-treated OC served as controls. Outcome analysis included immunohistochemical labelling of ribbon synapses, histochemical analysis of free reactive oxygen species and morphological analysis of stereocilia bundles. Overall, the protective effects of dexamethasone on electrically induced damage in cochlear explants were moderate. High-dose dexamethasone protected bundle integrity at higher current levels. Low-dose dexamethasone tended to increase ribbon density in the apical region.

## Introduction

Worldwide, approximately 466 million people suffer from disabling hearing loss (World Health Organization 2019). In cases of severe hearing impairment or deafness, the treatment of choice is cochlear implantation (CI). The concept of combined electro-acoustic stimulation (EAS) utilizes acoustic stimulation for the residual hearing in the low frequency range and direct electric stimulation of auditory nerve fibres for hearing restoration in the high frequency range (von Ilberg et al. 1999). Many different studies have shown that combined EAS in patients with residual hearing results in superior pitch perception, speech perception (in quiet and noise) and even better music perception when compared to electrical or acoustical stimulation alone (Turner et al. 2004; von Ilberg et al. 2011; Irving et al. 2014). Unfortunately, in a subset of patients, the residual hearing is lost months to years after CI insertion (Santa Maria et al. 2013; Skarzynski et al. 2013; Helbig et al. 2016). The structural and molecular mechanisms leading to the loss of residual hearing are currently unknown. Initial evidence suggests that electrical current could cause the loss of residual hearing (Reich et al. 2015; Al-Zaghal et al. 2019). In our own previous studies, we showed a reduction in neurite length and a decrease in spiral ganglion neuron (SGN) survival induced by high charge densities during electrical stimulation (Peter et al. 2019a). Additionally, a possible decrease in ribbon synapses at high charge densities was demonstrated (Peter et al. 2019b).

In order to reduce the consequences of CI insertion trauma such as inflammation and fibrosis, various glucocorticoids are already in clinical use and applied either systemically or locally during cochlear implantation (Skarżyńska et al. 2018; Prenzler et al. 2018). The protective effects of steroids have been evaluated in several systems and are potentially dose-dependent. In one study, application of dexamethasone at a concentration of 1 nmol/L was sufficient to improve the survival of SGN (Bohl et al. 2012). Dexamethasone has been used for otoprotection (Bird et al. 2011; Prenzler et al. 2018) and acts as a catalytic radical scavenger that counteracts oxidative stress (Bas et al. 2012; Sies 2015). Oxidative stress is induced by an overproduction of reactive oxygen species (ROS), which are a by-product of mitochondria in natural cellular respiration (Chance et al. 1979) or by inflammatory influences (Barnes 1990), cardiovascular disease (Cai and Harrison 2000) or ageing-related processes (Jiang et al. 2007). Similarly, oxidative stress plays a major role in hearing loss induced by noise, cisplatin and treatment with aminoglycoside antibiotics (Choi and Choi 2015; Sheth et al. 2017; Tavanai and Mohammadkhani 2017). Hydroxyl radicals, hydroperoxyl radicals and hypochlorite and superoxide anions are mentioned as examples of ROS. We aimed to determine whether steroids have the potential to avoid or reduce the damage that is induced by electrical stimulation. Organ of Corti explants were treated in vitro with two different concentrations of dexamethasone and subsequently electrically stimulated. We then determined the number of remaining synaptic ribbons and the level of free radical production. Since aminoglycoside toxicity and sound trauma can cause subtle changes in stereocilia, we evaluated inner hair cell stereocilia bundle morphology using stimulated emission depletion microscopy (StED) (Daudet et al. 1998; Bahloul et al. 2009).

## Materials and Methods

### Ethical approval

All applicable international, national and/or institutional guidelines for the care and use of animals were followed. This article does not contain any studies with human participants performed by any of the authors. All experiments were carried out according to the German “Law on Protecting Animals” (§4) and with the European Directive 2010/63/EU for protection of animals used for experimental purposes. These experiments are registered (No. 2016/118) with the local authorities (Laboratory Animal Science).

### Preparation and Tissue Culture

Petri dishes (Ø 35 mm, TPP, Switzerland) were provided with a grid for orientation on the outside of the bottom of the dish. Round coverslips (Ø 18 mm, OMNILAB, Germany) with four fixation points were attached to the Petri dishes. For the fixation points, a pen (ImmEdge Hydrophobic Barrier PAP Pen, Vector Laboratories, USA) containing a hydrophobic liquid suitable for cell culture was used. The coverslip was centrally aligned. The finished construct was treated for surface disinfection with ultraviolet radiation (Spectrolinker XL-1000 UV Crosslinker, Spectroline) for 30 min. Subsequently, the coverslips were coated with a 1 × 10^− 4^% poly-l-lysine solution (# P4707, Sigma-Aldrich, USA) for 30 min at 37 °C (Peter et al. 2019a). Finally, the Petri dishes were filled with 2 ml of medium and left in the incubator for at least 30 min: DMEM-F12 (Thermo Fischer Scientific, USA), 10% foetal calf serum (FBS Superior, Merck, Germany), 6 ppm glucose (B. Braun, Germany), 20 μg/mL insulin (# I0516, Sigma-Aldrich), 100 U/mL penicillin (# 1502701, Sigma-Aldrich) and 24 ng/mL of an insulin-like growth factor (# I8779, IGF-1, Sigma-Aldrich). For each experiment, two of the Petri dishes were supplemented with 1 nmol/L dexamethasone (Sanofi, France) and the other two Petri dishes with 1 μmol/L dexamethasone.

Preparation of explants followed our previously described protocol (Peter et al. 2019a). In brief, postnatal Sprague-Dawley rats (P2–4) of both sexes were decapitated and the petrous bone was removed from the skull. Subsequently, the membranous cochlea was freed from the bony cochlea and most of the *modiolus* and the *stria vascularis* were removed. The membranous cochlea was divided into three parts: apical, medial and basal. The apical and basal turns were transferred to the previously prepared, medium-filled dishes with the basilar membrane attached to the glass and the stereocilia facing the surface of the medium. Subsequently, the turns were positioned in a radius of 1–5 mm by means of the grid located on the dish. For adhesion to the coated glass surface, the sections of the cochleae were incubated at room temperature for 3 min and finally at 37 °C and 5% CO_2_ for 10 min (HeraCell VIOS 160i, Thermo Fisher Scientific). Finally, the cultures were incubated for 24 h at 37 °C and 5% CO_2_.

### Electrical Stimulation

The samples were electrically stimulated for 24 h, while the unstimulated references remained without an electrode in the incubator. The electrical stimulation was carried out as previously described (Peter et al. 2019a, b). A stimulation electrode made of biocompatible platinum/iridium wire (O’Malley et al. 2017) was positioned centrally on the coverslip with the tip touching the glass surface. The outer ring electrode was positioned on the bottom of the glass Petri dish. A charge-balanced biphasic rectangular pulse was used for electrical stimulation with the charge density decreasing with increasing radius. Cultures were stimulated at 0.44 and 2.0 mA to simulate a safe and an unsafe current level. The pulse width was set at 400 μs and the interpulse delay at 120 μs with a repetition rate of 1 kHz. This resulted in charge densities of 1.1 to 17.9 μC cm^− 2^ phase^− 1^.

### Immunocytochemical Staining and Microscopic Image Acquisition

After electrical stimulation, the explants were incubated with 5 μmol/L of CellROX Deep Red (# C10422, Thermo Fischer Scientific) (30 min at 37 °C, 5% CO_2_). CellROX Deep Red starts to fluoresce in the cytoplasm when oxidized, yielding a measure of ROS production. Subsequently, the samples were rinsed three times with Ca^2+^/Mg^2+^-free d-phosphate buffer (PBS, Thermo Fischer Scientific). Finally, the sections were fixed with a 4% paraformaldehyde solution (Merck) and also rinsed three times with PBS. Immunofluorescent labelling of presynaptic ribbon synapses was carried out using rabbit anti-CtBP2 (1:100, # CG1809, Cell Applications, USA) and anti-rabbit DyLight 549 (1:500, # 711-505-152, Jackson ImmunoResearch, UK). Actin in stereocilia was labelled with phalloidin coupled with Alexa Fluor 488 (1:40, # A12379, Thermo Fischer Scientific). ProLong™ Gold (# P36934, Thermo Fischer Scientific) was applied to the finished sections of the cochlea which were then cover-slipped (type #1, thickness 0.13–0.16 mm, Menzel-Gläser, Germany).

The microscopic images were captured with a confocal laser scanning StED microscope (TCS SP8 X, Leica, Germany). For each object, a *z*-stack of 20 images with a height of 1 ± 0.5 μm was taken and finally assembled into a raw image. The objective used was a × 100 (HCX PL APO × 100/1.40 OIL, Leica) with immersion oil (type F, Leica). For fluorescence imaging, a 592-nm depletion laser and a white-light laser were used. For the representation of the ribbons, the confocal mode was used, and for the phalloidin label, the StED mode was used. The laser power was kept constant in all fluorescence images. For the semi-automatic evaluation of the ribbon number, an algorithm (synapse counter (Dzyubenko et al. 2016)) was used for ImageJ (National Institutes of Health, USA). For this purpose, a range comprising of six inner hair cells was selected from the raw images in order to subsequently automatically count the marked ribbons with a predefined threshold value. The threshold “max entropy” selected in ImageJ overlays a mask over the six inner hair cells, increasing the entropy in the image. This technique analyses the whole stack histogram of a series of images to maximize the acquisition of signals of interest. The remaining image parts were counted using the previously determined minimum and maximum areas for the ribbon synapses. In representative images of the organ of Corti, all ribbon synapses were counted and then divided by the number of the depicted inner hair cells. As a result of this, the ribbon number per inner hair cell (also called the ribbon density) was determined. The ribbon density of the electrically stimulated samples was set in relation to the ribbon density of the unstimulated references to finally obtain the relative ribbon density as previously described (Peter et al. 2019b).

**Fig. 1:**
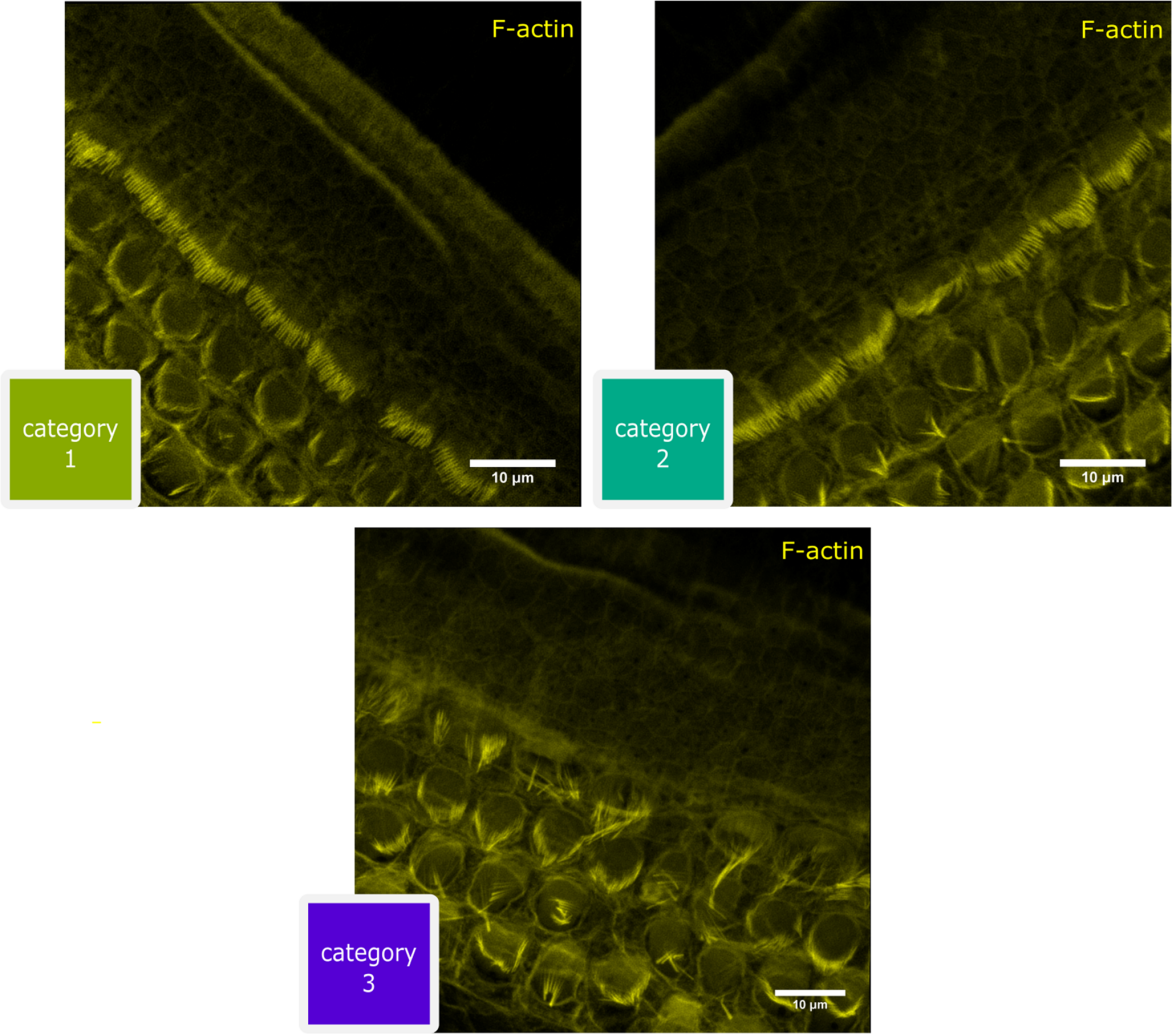
Classification of the stereocilia bundles of the inner hair cells. Category 1: organized stereocilia; all cilia are ordered and have the same orientation. Category 2: partially disorganized stereocilia; partially, some inner hair cells have bundles that show a different orientation (arrow). Category 3: completely disorganized stereocilia; the majority of the stereocilia of the inner hair cells has no uniform orientation and ordered bundles are not seen. Scale bar = 10 μm

For the detection of reactive oxygen species, the intensity of the fluorescence marker CellROX Deep Red was measured in the same six inner hair cells. For this purpose, a constantly uniform rectangle (ROI = 200 × 1000 pixels) was placed over the area of the six inner hair cells using ImageJ, in which the ribbon synapses were localized. For this range, the mean fluorescence intensity was determined and was related to the unstimulated references. Inner hair cell stereocilia bundles were classified by the level of organization as shown in Fig. 1. The categories follow the increasing disorder of the stereocilia and were subjectively determined for each StED image: category 1 is organized stereocilia, i.e. if more than 70% of inner hair cells showed completely organized stereocilia; category 2 is partially disorganized stereocilia, i.e. 50–70% of inner hair cells with completely organized stereocilia; and category 3 if more than 50% of inner hair cells showed completely disorganized stereocilia. Each cell of the same six inner hair cells as described above was classified into one of the three categories.

### Statistical Evaluation

The experiments with a current of 0.44 mA were carried out with *N* = 8 repetitions and the experiments with 2.0 mA with *N* = 5. The number of samples evaluated is indicated by the variable *n*. The results were presented as the mean $\overline{x}$ ± standard error of the mean (SEM). The software (GraphPad, USA) was used for the statistical evaluation of the data. For the determination of the significances, a one-way ANOVA with a connected Tukey multiple comparison was performed and an error probability of 5% = *p* < 0.05 was assumed. If the number of samples was too small, a *t* test with the same error probability was used. For the statistical analysis of the data, a normal distribution was assumed, because with such a small amount of data, a Kolmogrov-Smirnov study does not guarantee statistical certainty.

## Results

### Structural and Molecular Alterations

**Fig. 2:**
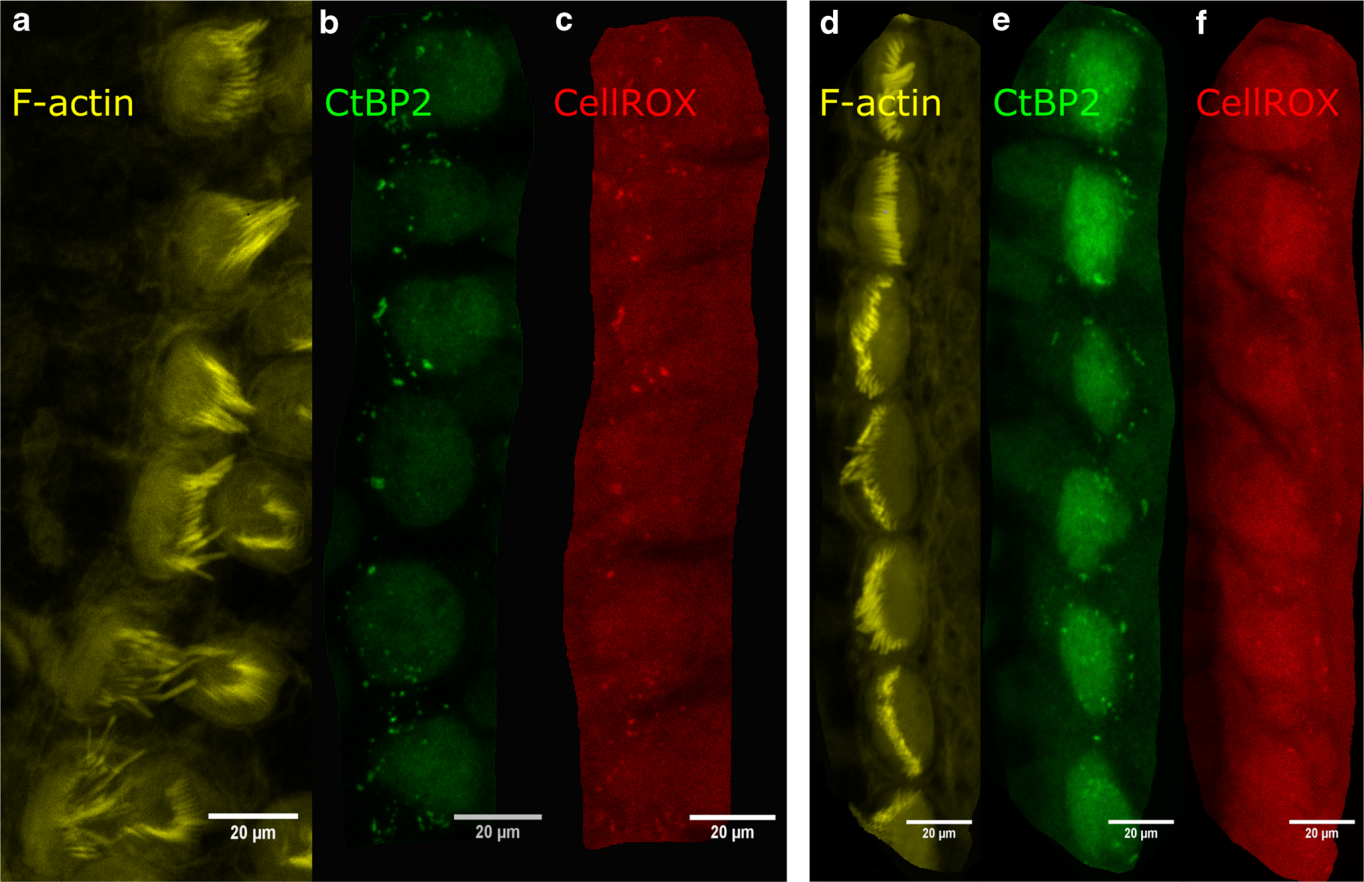
Fluorescent images of a 2-mA electrically stimulated (**a–c**) and an unstimulated (**d–f**) basal region of the cochlea with 1 nmol/L dexamethasone in the culture medium. **b**, **c**, **e** and **f** were displayed in confocal mode, whereas **a** and **d** were recorded in StED mode. **b**, **e** Green indicates the ribbon synapses with CtBP2 and **c**, **f** the red colour indicates the oxidation products of CellROX Deep Red. **a**, **d** Stereocilia F-actin was labelled with phalloidine. Scale bar 20 μm

To screen for structural and metabolic changes induced by electrical stimulation, stimulated (Fig. 2a–c) and non-stimulated (Fig. 2d–f) organ of Corti explants were evaluated after triple labelling (stereocilia, ribbons and ROS). Inner hair cell stereocilia were stained with phalloidin in yellow (Fig. 2a, d). Inner hair cells cultured under electrical stimulation clearly displayed disorganized stereocilia when compared to the unstimulated control. Examples of ribbon staining are shown in panels b and e and CellRox labelling is shown in panels c and f. Interestingly, CtBP2 labelling and CellROX intensity overlapped in electrically stimulated hair cells (Fig. 2b).

### Electrical Stimulation Increases Disorganization of Inner Hair Cell Stereocilia

**Fig. 3:**
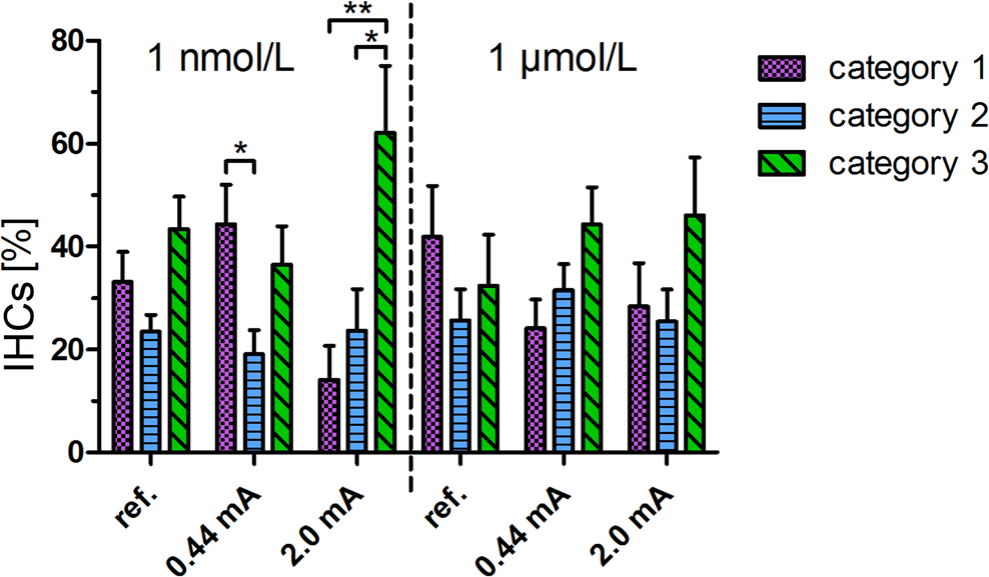
Stereocilia bundle morphology varies with the stimulus amplitude used. The unstimulated references and stimulated samples presented here were derived from apical and basal regions of the cochlea. Category 1: organized stereocilia; category 2: partially disorganized stereocilia; category 3: completely disorganized stereocilia. **p < 0.01, *p < 0.05

The electrically stimulated organ of Corti explants were treated with dexamethasone at two different concentrations in order to evaluate a dose-dependent protective effect after steroid treatment. Based on our classification system (1 = organized stereocilia; 2 = partially disorganized stereocilia; 3 = completely disorganized stereocilia), the percentage of completely disorganized stereocilia under electrical stimulation with 2 mA and 1 nmol/L dexamethasone was significantly higher (**p < 0.01; Fig. 3). The percentage of unorganized stereocilia decreased from 62 to 46% as the dose of dexamethasone was increased.

The unstimulated controls showed an equivalent mix of all categories in both dexamethasone concentrations. For unstimulated controls with 1 nmol/L dexamethasone, a distribution of 33% (category 1), 24% (category 2) and 43% (category 3) was determined. At low doses of dexamethasone, higher levels of current induced a significant increase in stereocilia bundle disorganization (category 3 **p < 0.01 in relation to category 1 and *p < 0.05 in relation to category 2). The incidence of category 3 stereocilia bundles seen at high current and high-dose dexamethasone was slightly but not significantly increased when compared to the unstimulated reference (46.1 ± 11.3% vs. 32.4 ± 9.9%; n.s.; Fig. 3).

### Quantification of Ribbons

**Fig. 4:**
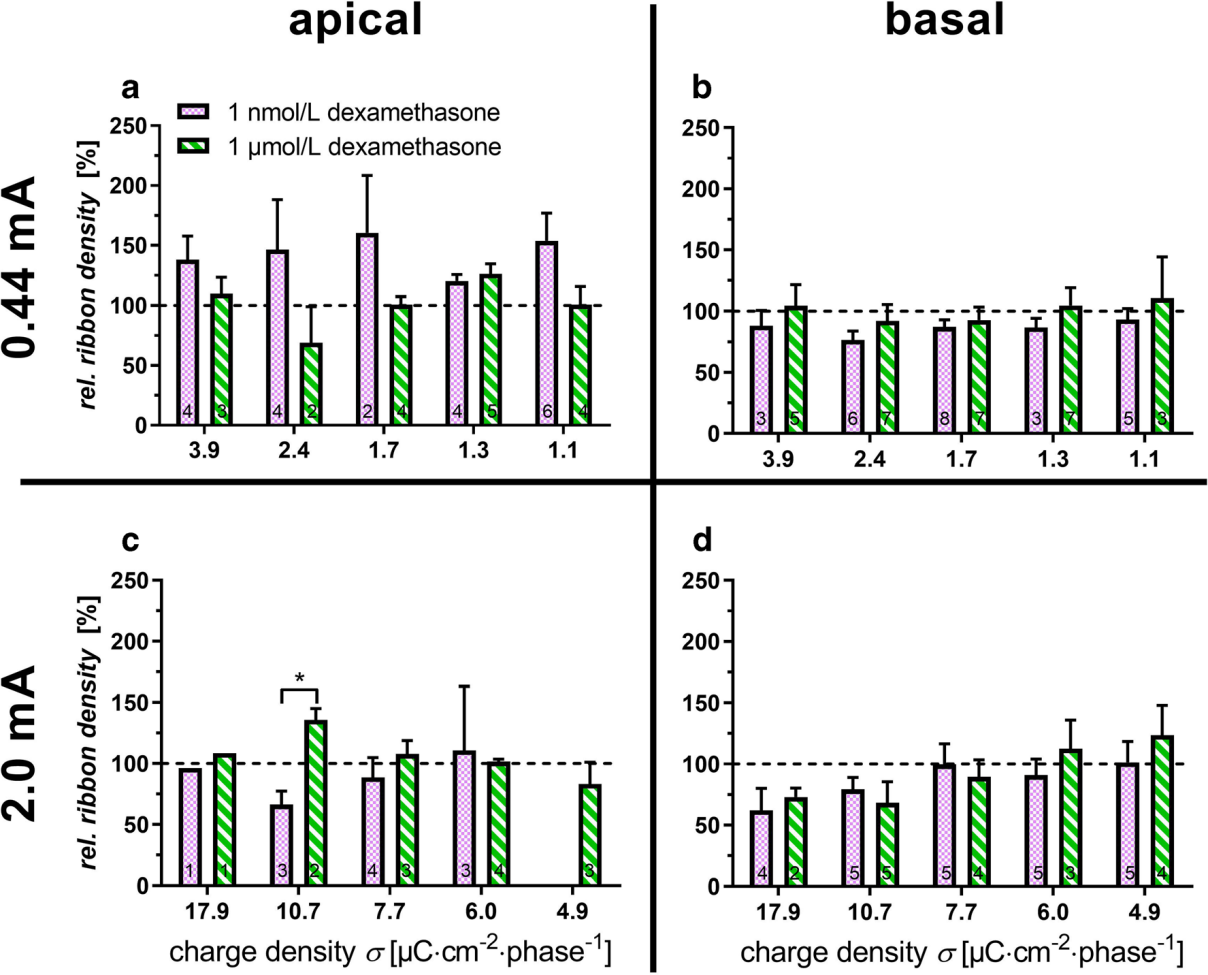
Relative density of ribbons with different dexamethasone concentrations. Analysis of the apical turn is depicted in **a** and **c** and of the basal turn in **b** and **d**. Low current level is depicted in **a** and **b** and high current level in **c** and **d**. A relative ribbon density of over 100% indicates an increased density, while below 100% indicates a decrease in density relative to the unstimulated references. The numbers in the columns represent the number of samples (*n*). The dashed line represents the underlying unstimulated reference. *p < 0.05

In previous studies, we were able to show that the number of ribbon synapses can degenerate due to an applied electric field. In the present study, a possible protective effect of dexamethasone on electrically induced degeneration of the ribbons was investigated. In the same six inner hair cells that were evaluated in terms of stereocilia, the ribbons were quantified. Also, the stimulated samples were compared to the unstimulated references. The ribbons on the apical and basal cochlear turns were quantified and were set in relation to the concentration of dexamethasone used (Fig. 4). When considering the apical turn and a dexamethasone concentration of 1 nmol/L, a potentially protective effect was observed at a current of 0.44 mA (Fig. 4a). When the concentration of dexamethasone in the apical region was increased to 1 μmol/L, a slight reduction of the ribbon density was observed at a charge density of 2.4 μC cm^− 2^ phase^− 1^. Overall, the lower levels of steroid resulted in an increased ribbon density.

Increasing the amplitude to 2 mA reduced the relative ribbon density in the apical region to an average of 90.5 ± 9.3% (mean overall four radii, Fig. 4c). At an amplitude of 2 mA, an increased concentration of dexamethasone resulted in an overall improved maintenance of the ribbons in the apical region compared to explants treated with 1 nmol/L dexamethasone. A significant increase in relative ribbon density was determined at a charge density of 10.7 μC cm^−2^ phase^−1^ and a concentration of 1 μmol/L dexamethasone.

Overall, the basal area of the cochlea shows less variation in ribbon density independent of charge densities and dexamethasone concentration. Low-level electrical stimulation (0.44 mA) in the basal area combined with low levels of dexamethasone only slightly changed the relative ribbon density. The relative ribbon density at 1 μmol/L dexamethasone was slightly higher than at 1 nmol/L. The difference between the two concentrations was at most 18% (charge density 1.3 μC cm^− 2^ phase^−1^). There was a significant difference between the apical and basal area at a charge density of 1.7 μC cm^−2^ phase^−1^ and 1 nmol/L dexamethasone. Thus, a significant (*p = 0.023) higher relative ribbon density was detected in the apical region at this charge density. Increasing the amplitude to 2 mA had a negative impact at a higher charge density of 10.7–17.9 μC cm^−2^ phase^−1^ on the ribbons (Fig. 4d). With decreasing charge density, however, ribbon densities approached control levels.

### Quantification of Oxidative Stress

**Fig. 5:**
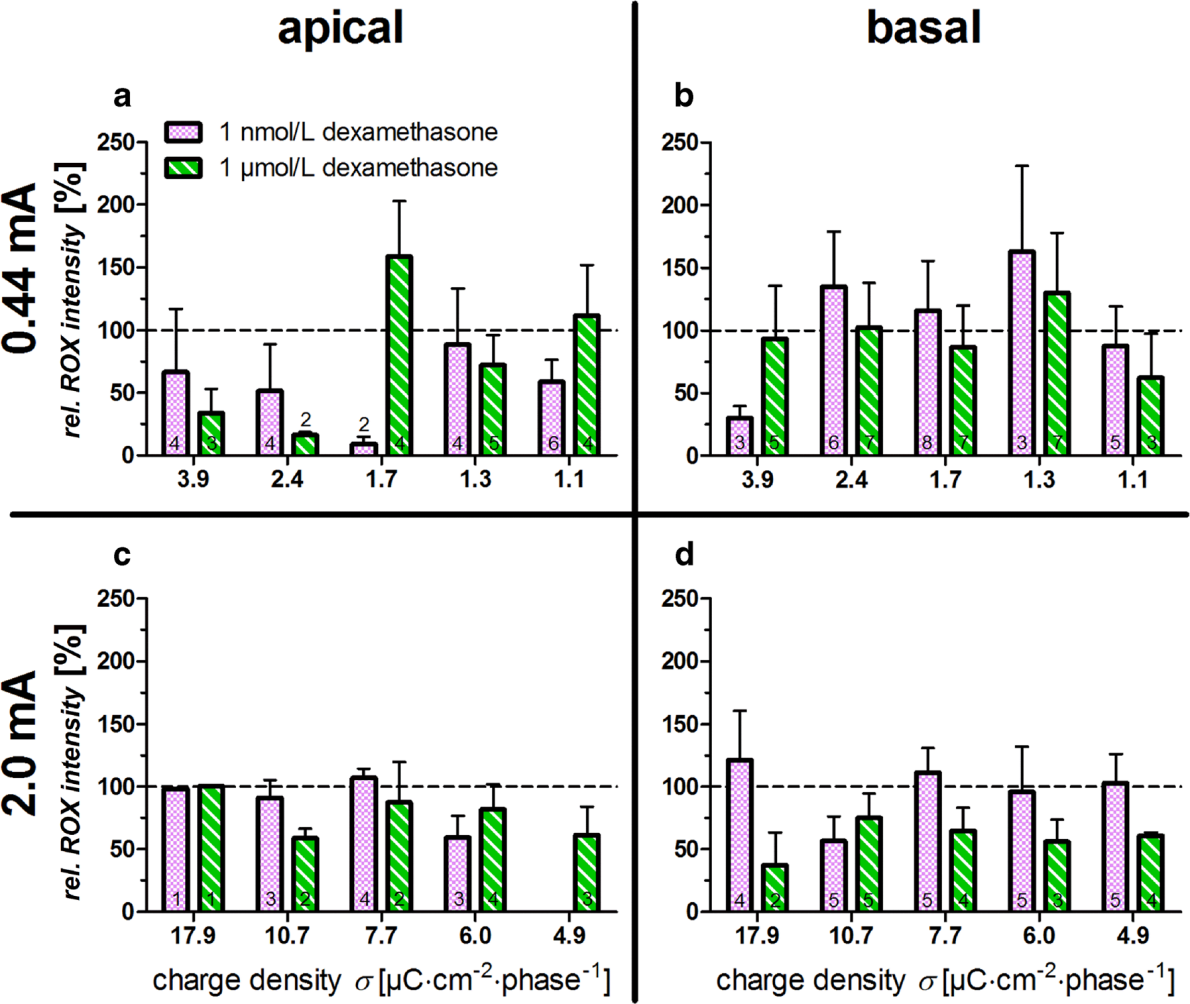
**a**–**d** Relative fluorescent intensity of the CellROX Deep Red Marker after application of electrical stimulation and different concentrations of dexamethasone. A relative intensity of over 100% indicates an increased accumulation of reactive oxygen species, while below 100% indicates a decrease in the reactive oxygen species relative to the unstimulated references. The numbers in the columns represent the number of samples (*n*). Dashed line represents the underlying unstimulated reference

The effect of electrical stimulation and dexamethasone on oxidative stress was evaluated with histochemical staining with CellROX. In the apical turn, at 0.44 mA and low dexamethasone concentrations, fluorescence levels were below control levels (Fig. 5a). Higher levels of oxidative stress were observed at 1.7 μC cm^− 2^·phase and high levels of dexamethasone. At low charge densities, a relative increase in CellROX intensity was observed in the basal turn (Fig. 5b). In the apical turn, higher current densities resulted in fluorescence levels similar to controls. As the charge density decreased to lower levels, CellROX intensity was reduced irrespective of the dexamethasone concentrations used (Fig. 5c). In the basal turn, high current levels and low dexamethasone dose generally showed CellROX levels similar to the unstimulated controls. High dose dexamethasone led to a reduced CellROX fluorescence intensity compared to controls (Fig. 5d).

## Discussion

Several studies have raised the possibility that electrical stimulation by a cochlear implant may have damaging effects on the inner ear under specific circumstances (Kopelovich et al. 2015; Reich et al. 2015; Peter et al. 2019b). Electrotoxicity could be responsible for the loss of residual hearing that may occur months or even years after cochlear implantation (Santa Maria et al. 2013; Skarzynski et al. 2013). The present study provides initial evidence that in vitro oxidative stress could be triggered by electrical stimulation and that the anti-inflammatory and radical scavenging substance dexamethasone can be partially protective. Indeed, a recent study showed that electrical stimulation increased ROS and reactive nitrogen species leading to loss of peripheral spiral ganglion neuron fibres (Liang et al. 2019).

In the herein presented study, P3 organ of Corti explants were exposed to safe and unsafe charge densities by varying current levels and distance to a central electrode as described previously (Peter et al. 2019a). We previously demonstrated that electrical stimulation significantly reduced survival of spiral ganglion cells and neurite length at higher charge densities (Peter et al. 2019a). Additionally, electrical stimulation was found to lead to a reduction in ribbon density in the inner hair cells (Peter et al. 2019b). Dexamethasone is an extensively investigated and frequently used anti-inflammatory drug (Vandewalle et al. 2018) that is currently being used to protect residual hearing in cochlear implant recipients (Skarżyńska et al. 2018). We demonstrate that adverse effects on inner hair cells induced by electrical stimulation could be partially prevented by dexamethasone. There were basal to apical differences in response to both electrical stimulation and dexamethasone dose. At the specific developmental time point (P3) chosen, the cochlea of the rat is immature. Hearing onset starts later at about P12. During development, there is a gradient in maturation between the apical and basal turn with the basal turn developing first (Booth et al. 2018). Our investigations were carried out in basal and apical fragments of the cochlea, and a difference in the degree of maturation might have an influence on the results of our study. However, differences in regional responses to trauma have been also demonstrated in adults in several studies (Johnson and Canlon 1994; Bohne and Harding 2000; Sha et al. 2001). Follow-up investigations on the influence of electrical stimulation and steroids should be performed in vivo in adult models to rule out the presence of different developmental stages along the cochlea.

**Fig. 6:**
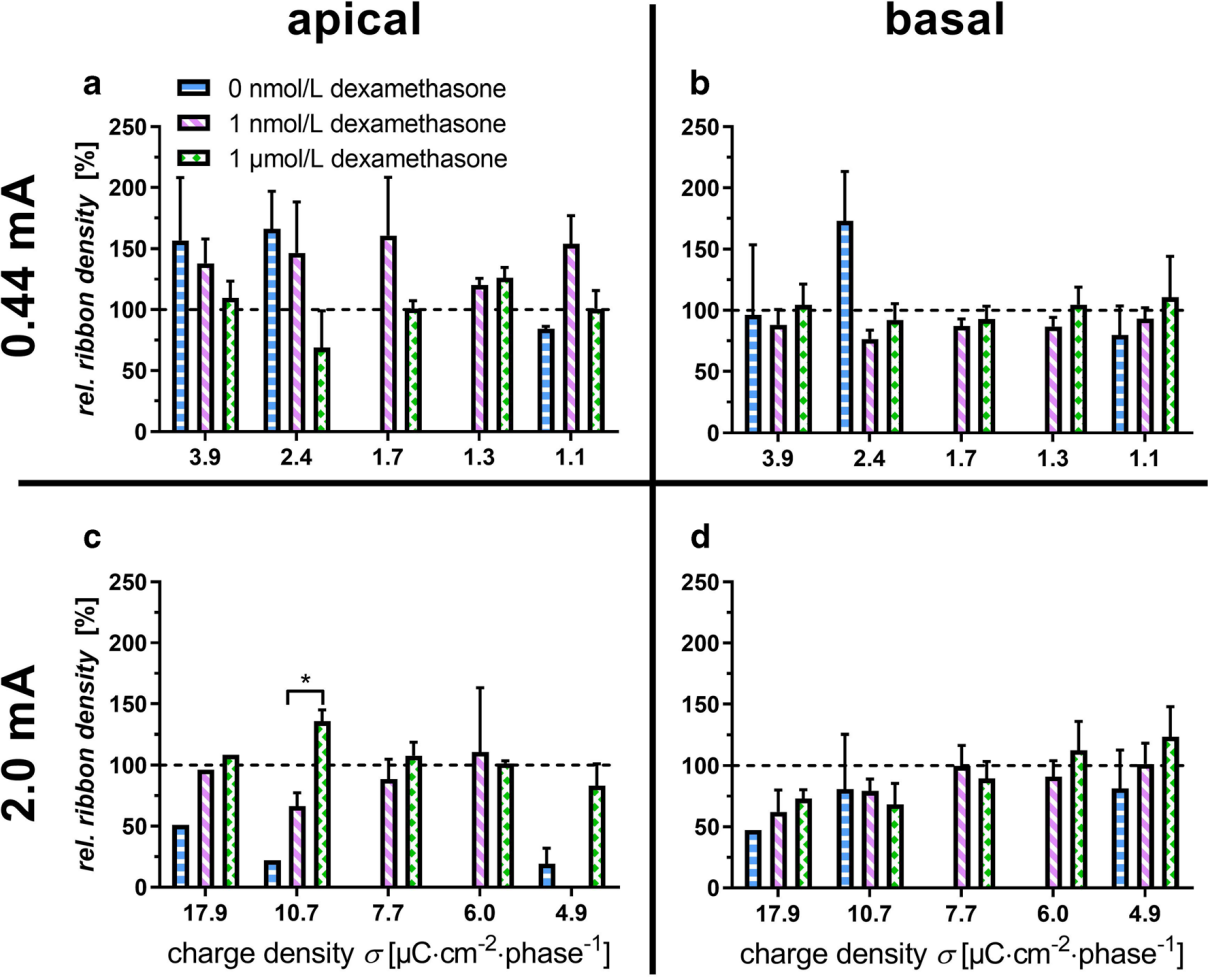
A comparison of the relative ribbon density with and without dexamethasone. The data without dexamethasone are from the study by Peter et al. 2019b. In the study without dexamethasone, not all charge densities were investigated (7.7 and 6.0 as well as 1.7 and 1.3 μC/cm^2^/phase were therefore not included). Without dexamethasone *N* = 3, with dexamethasone 0.44 mA *N* = 8, 2.0 mA *N* = 5

Disorganization of the inner hair cell stereocilia bundles may be one of the initial signs of structural damage and is more likely to occur after electrical stimulation (Fig. 3). Disorganized stereocilia bundles are also seen after aminoglycoside ototoxicity and noise trauma (Daudet et al. 1998; Bahloul et al. 2009). However, our study is the first to report about disorganized stereocilia bundles as a consequence of electrical stimulation. We compared the results from our current study to our previous work (Peter et al. 2019a). As can be seen in Fig. 6, dexamethasone seems to prevent the loss of the ribbons in the apical and basal regions induced under electrical stimulation at high charge densities (> 4.9 μC cm^− 2^ phase^− 1^). Electrical stimulation without treatment with dexamethasone at a charge density of 17.9 μC cm^− 2^ phase^− 1^ and 2 mA in the apical turn resulted in a reduced relative ribbon density of 51.1% when compared to the treatment with 1 nmol/L dexamethasone at the same charge density (96.3% relative ribbon density).

The significant difference in Fig. 4c to higher relative synapse densities at 1 μmol/L dexamethasone and 2 mA is also reflected by a decrease in ROX at a charge density of 10.7 μC cm^− 2^ phase^− 1^ (Fig. 5c). There is a tendency for the relative intensity of ROX to decrease over the 4.9–10.7 μC cm^− 2^ phase^− 1^ in the apical region. At lower charge densities (< 4.9 μC cm^− 2^ phase^− 1^), a putative protective effect was seen (Fig. 5a). At 0.44 mA and 1 nmol/L in the apical turn at a charge density of 3.9 μC cm^− 2^ phase^− 1^, the relative ribbon density with 1 nmol/L dexamethasone (138.0 ± 19.7%) was comparable to the one obtained for the stimulated samples without dexamethasone (156.5 ± 51.6%) (Peter et al. 2019b). Indeed, there are differences observed in the behaviour of the two different cochlear regions with apical inner hair cells being more robust at higher charge densities and electrical stimulation being even beneficial especially for the inner hair cells in the apical turn. Studies have shown that the basal part of the organ of Corti is particularly sensitive to trauma and that there are differences in gene expression along the length of the organ of Corti (Aran et al. 1982; Ladrech et al. 2007; Booth et al. 2018).

Glucocorticoids have a pleiotropic range of effects. They alter gene expression but also have the potential to trigger responses that do not depend on genomic changes. These responses can be dose-dependent. At high concentrations (e.g. 1 g/day methylprednisolone used in the treatment of multiple sclerosis), the physiological effects of steroids are mediated via the cell membrane-bound glucocorticoid receptors as well as via membrane stabilization. Effects are seen within a period of seconds to minutes (Finsterer and Frank 2014). In contrast, low-dose glucocorticoids (e.g., in cerebral vasculitis 1 mg day^− 1^ kg^−1^ body weight prednisolone) act over a period of 30 min to 8 h via the trans-expression or transactivation of the cytosolic glucocorticoid receptor NR3C1 (Finsterer and Frank 2014). Dexamethasone also inhibits prostaglandin release (Lewis et al. 1986), thus reducing the inflammatory response. In the study presented herein, a genomic mechanism of action could be expected due to the applied dexamethasone concentrations ranging from 5 pg to 5 fg. In humans, the highest concentration of glucocorticoid receptors required for the action of dexamethasone are mainly located in the spiral ligament (Rarey and Curtis 1996; Lee et al. 2019) and increase from basal to apical in type I, II and V fibrocytes (Kil and Kalinec 2013). In addition, glucocorticoid receptors are also located in inner and outer hair cells as well as in the auditory nerve (Lee et al. 2019). Understanding the exact mode of action would give us important information about the time frame before or after electrical stimulation in which treatment should have been initiated. In addition, the mode of application is also important. For example, after transtympanic delivery, dexamethasone was rapidly cleared from the inner ear. If a prolonged treatment with steroids is required to prevent electrotoxicity, local delivery may not be adequate (Parnes et al. 1999). In our study, we could show that the combination of a 24-h pre-treatment with dexamethasone solution prior to electrical stimulation could mitigate some of the effects of electrical stimulation. However, detrimental effects of steroids have also been reported and need consideration while interpreting our results. For example, glucocorticoid-receptor-mediated stress hormone signalling has a negative impact on auditory nerve responses, inner ear synapses and central auditory processing without affecting the hearing threshold in an animal model of noise trauma (Singer et al. 2018). Neurotoxicity due to steroids has been already described for other organ systems (Dumas et al. 2010; Pomara et al. 2015; Roy and Sapolsky 2003). Glucocorticoids inhibit the re-uptake of excitotoxic glutamate from the synapse, impair calcium regulation and increase accumulation of oxygen radicals (Stein-Behrens et al. 1992), thereby inducing excitotoxicity (Roy and Sapolsky 2003; Sorrells et al. 2014) and compromising neuronal survival (Kaufer et al. 2004). Other potential deleterious effects of glucocorticoids include impaired wound healing (Carolina et al. 2018).

Our experimental approach has some limitations. Only the apical and basal regions of the organ of Corti were examined to maximize the regional differences in the response to treatment. To model loss of residual hearing, evaluation of the apical turn of the cochlea is vital since this is the region of the inner ear where residual hearing/hair cells are most often seen. The use of neonatal explants (postnatal day 3) also limits our ability to evaluate the neural (post synaptic) ribbon synapse since these are being dynamically remodelled at this stage of development (Coate et al. 2019). Overall, we evaluated a lower number of apical turns than basal turns. This is related to the anatomy of the cochlea and the degree of coiling. The apical turn has a reduced contact area with a segment protruding in the medium without any contact to the bottom of the dish. Due to this, the samples of the apical regions are more exposed to hydrodynamic influences, e.g. during transportation from the microscope to the incubator. Thus, many apical parts detach from the bottom of the culture dish and are incubated as floating cultures that are lost in the process of discarding the medium and fixation. Precise control of electrical stimulation is not possible with floating cultures. Increasing the number of measurements may be necessary to delineate possible statistically significant alterations due to electrical stimulation and/or treatment with steroids.

The labelling of the reactive oxygen species in the organ of Corti often coincided with the CtBP2-labelled ribbon synapses (Fig. 2). An interference of the emission bands of both dyes can be excluded, since the filter limits in the microscope were exactly adjusted with the aid of an acousto-optical modulator and thus separated from other emission bands. Oxidation of individual components of the ribbons could indicate potential damage to the inner hair cells. Mitochondria (Bullen et al. 2015), which are more commonly located at the base of the hair cells in the vicinity of the ribbon synapses, could release more reactive oxygen species, thereby damaging the synapses. This assumption is corroborated by a recent study where a pharmacological upregulation of endogenous antioxidative enzymes was able to rescue changes induced by electrical stimulation (Liang et al. 2019). Labelling of the postsynaptic receptors would have been helpful in assessing damage to synaptic signalling. However, glutamate receptors cannot be successfully labelled in neonatal rats at the age of P2–4. These are only assembled at the age of 8 days (Knipper et al. 1996; Safieddine et al. 2012; Liberman and Liberman 2016). A further study demonstrated that targeted excitotoxicity in vitro by NMDA receptor agonists and kainic acid (a glutamate analog) caused a reduction in pre- and post-synaptic labelling of the organ of Corti (Wang and Green 2011). In parallel, no significant changes were found in the hair cells or the SGN (Wang and Green 2011). This and the detection of reactive oxygen species could indicate that changes at the synaptic level occur first, and thereafter, damage becomes visible at the cellular level.

In this study, we demonstrate that dexamethasone may partially stabilize ribbon synapses and may inhibit oxidative stress induced by electrical stimulation. High-dose dexamethasone protected bundle integrity at higher current levels. Low-dose dexamethasone tended to increase ribbon density in the apical region. However, marked differences in the cochlear turns were also observed indicating that the apical region could be more responsive to steroid treatment than the basal turn. The altered organization of the stereocilia after electrical stimulation could be used as another damage marker.
